# Hydrogen Sulphide Production in Healthy and Ulcerated Gastric Mucosa of Rats

**DOI:** 10.3390/molecules22040530

**Published:** 2017-03-27

**Authors:** Patrycja Bronowicka-Adamska, Maria Wróbel, Marcin Magierowski, Katarzyna Magierowska, Sławomir Kwiecień, Tomasz Brzozowski

**Affiliations:** 1 Chair of Medical Biochemistry, Jagiellonian University Medical College, Krakow, 7 Kopernika St., 31-034 Cracow, Poland; mbbronow@cyf-kr.edu.pl; 2 Department of Physiology, Jagiellonian University Medical College, 31-530 Krakow, Poland; magierowskim@yahoo.pl (M.M.); k.jasnos@interia.pl (K.M.); skwiecien@cm-uj.krakow.pl (S.K.); mpbrzozo@cyf-kr.edu.pl (T.B.)

**Keywords:** hydrogen sulphide, gastric mucosa, NaHS, cystathionine-β-synthase, 3-mercaptopyruvate sulfurtransferase, rhodanese, γ-cystathionase

## Abstract

Hydrogen sulphide (H_2_S) is produced endogenously via two enzymes dependent on pyridoxal phosphate (PLP): cystathionine beta-synthase (CBS, EC 4.2.1.22), cystathionase γ-liase (CTH, EC 4.4.1.1), and a third, 3-mercaptopyruvate sulfurtransferase (MPST, EC 2.8.1.2). H_2_S strengthens the defence mechanisms of the gastric mucosal barrier, and plays an important role in gastroprotection, including the increased resistance to damage caused by various irritants and non-steroidal anti-inflammatory drugs. The study was conducted to determine the role of H_2_S in ulcerated gastric mucosa of rats caused by immobilization in cold water (WRS). The activity and expression of γ-cystathionase, cystathionine β-synthase, 3-mercaptopyruvate sulfurtransferase, and rhodanese was compared with healthy mucosa, together with H_2_S generation, and cysteine, glutathione, and cystathionine levels. The results showed that the defence mechanism against stress is associated with stimulation of the production of H_2_S in the tissue and confirmed the observed advantageous effect of H_2_S on healing of gastric ulcers. In case of animals pretreated with exogenous sources of H_2_S and NaHS, and some changes observed in the ulcerated gastric mucosa tend to return to values found in the healthy tissue, a finding that is in accordance with the previously determined gastroprotective properties of H_2_S. The results presented in this paper point to the possible role of rhodanese in H_2_S production in the gastric mucosa of rats, together with the earlier mentioned three enzymes, which are all active in this tissue.

## 1. Introduction

**Scheme 1:**
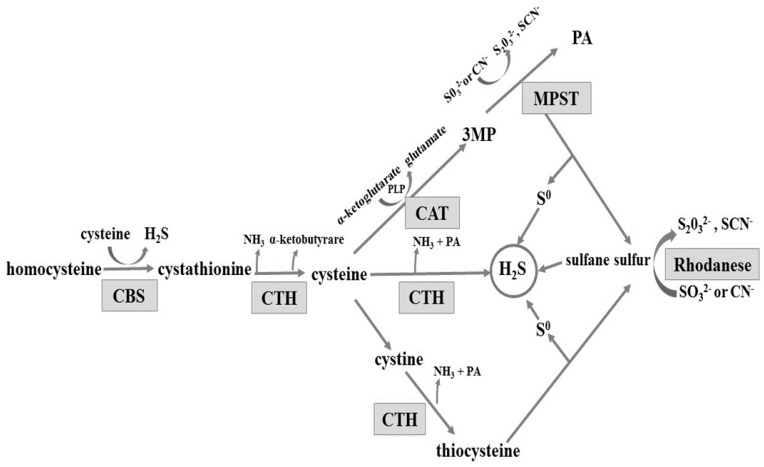
Enzymatic pathways involved in hydrogen sulphide formation. CBS: cystathionine β-lyase; CTH: γ-cystathionase; MPST: 3-mercaptopyruvate sulfurtransferase; CAT: cysteine aminotransferase; 3MP: 3-mercaptopyruvate; PA: pyruvate; and PLP: pyridoxal phosphate; CN^−^: cyanide; SCN^−^: thiocyanate.

The gastrointestinal tract is exposed to various substances and factors which often cause gastric mucosal damage. Long-term exposure to these factors, or to stress, can result in pathological inflammation, such as erosions, haemorrhages, or ulcers. An important role in maintaining the integrity of the gastric mucosa is played by hydrogen sulphide (H_2_S). H_2_S is produced from l-cysteine enzymatically in pathways involving three enzymes: cystathionine β-synthase (CBS, EC 4.2.1.22), cystathionine γ-lyase (CTH, EC 4.4.1.1), and 3-mercaptopyruvate sulfurtransferase (MPST EC 2.8.1.2) (reviewed in [1]). CBS and CTH are enzymes dependent on pyridoxal phosphate (PLP). The MPST reaction converts 3-mercaptopyruvate produced from cysteine in a PLP-dependent transamination reaction (Scheme 1). CBS produces cystathionine and H_2_S from l-cysteine or homocysteine. Cystathionine is converted by CTH to l-cysteine, alpha-ketobutyrate, and ammonia. Cysteine can be also converted by CTH to pyruvate and H_2_S. Alternatively, after oxidizing to cystine, it is converted by CTH to thiocysteine—a sulfane sulfur-containing compound. The third enzyme, 3-mercaptopyruvate sulfurtransferase, acts in combination with cysteine aminotransferase (CAT, EC 2.6.1.3) (Scheme 1). Cysteine aminotransferase catalyses l-cysteine transamination to 3-mercaptopyruvate (3MP). MPST catalyses the transfer of sulphur atom from 3MP to sulphite and the product of this reaction, thiosulfate, can be further reduced to hydrogen sulphide. Non-enzymatic reduction of sulfane sulphur to hydrogen sulphide can occur in the presence of glutathione (GSH) [2]. In the mitochondria, hydrogen sulphide is oxidized to sulphite, which is then converted to thiosulfate (a sulfane sulphur-containing compound) by thiosulfate sulfurtransferase (rhodanese; TST, EC 2.8.1.1) [1,3,4,5] (Scheme 1). Thus, rhodanese can be involved in H_2_S generation in the mitochondria [6].

Stress in the gastrointestinal tract may affect the motility, secretion of glands, the mucous membrane, or the flow in small blood vessels. Water immersion restraint stress (WRS) of rats are commonly used for studying stress-induced gastrointestinal erosion and ulcers [7]. Experiments carried out by Lou et al. [8] on an animal model of stress induced by immersing the animals in water and their fixation at a low temperature showed that H_2_S reduced the amount of gastric mucosal damage and statistically significantly reduced the concentration of lipid peroxidation compared to the control group exposed only to the stress from the cold and immobilization.

The study was conducted to determine the role of H_2_S in the inflammatory process associated with damage to the gastric mucosa of rats caused by stress and immobilization in cold water (WRS). The investigations involved the comparison of the activity and expression of CTH, CBS, MPST, and TST in the gastric mucosa of rats not exposed and exposed to WRS. H_2_S generation, and cysteine, glutathione and cystathionine levels were also compared. The results showed an increased production of H_2_S as a defence against damage of the gastric mucosa caused by WRS. NaHS, a donor of H_2_S, administered to animals before WRS, resulted in the reversion of some investigated parameters to values found in healthy tissue, thus confirming the gastroprotective properties of H_2_S.

## 2. Results and Discussion

**Figure 1:**
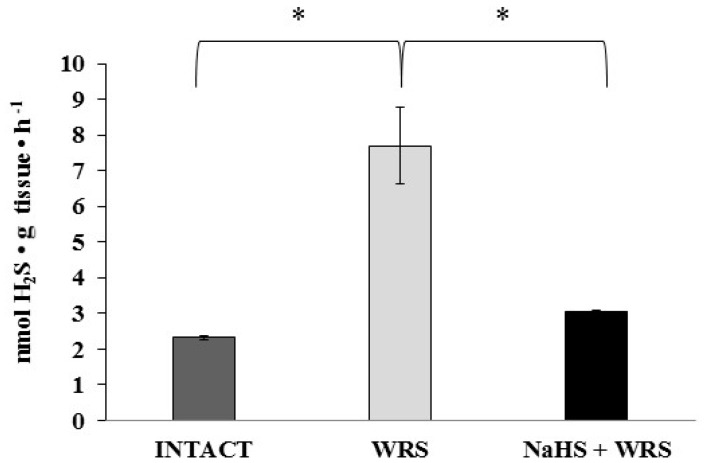
The level of hydrogen sulphide after 1 h of incubation with l-cysteine in the experimental group. The experiments were carried out in gastric mucosa homogenates in three experimental groups (Intact, water immersion restraint stress (WRS), WRS + NaHS), with l-cysteine as a main endogenous substrate for the hydrogen sulphide-producing enzymes. The data represent the mean value from two independent experiments. Statistical analysis was performed using the Student’s *t*-test (* *p* < 0.05).

The studies were conducted in the gastric mucosa from healthy rats and rats with ulcers induced by immersion in cold water (21 °C) for 3.5 h (WRS). Differences observed in hydrogen sulphide generation (Figure 1) confirm the beneficial effect of H_2_S in ulcer healing in rats [9].

### 2.1. H*_2_*S Generation in Gastric Mucosa of Healthy Rats

**Figure 2:**
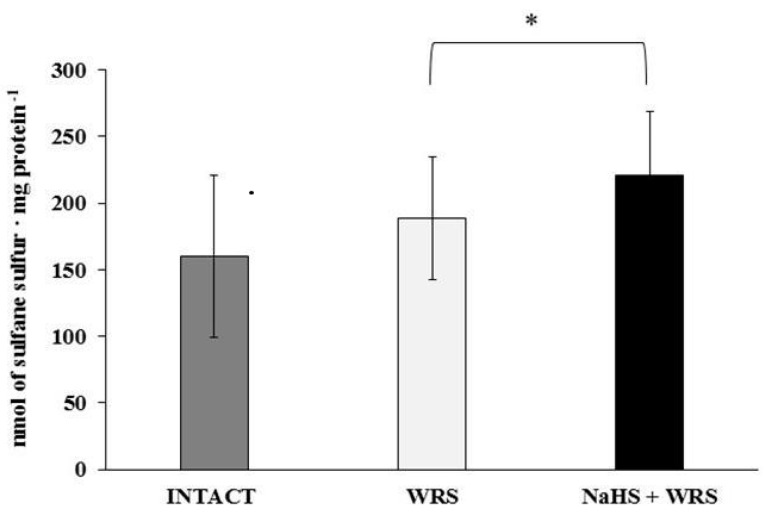
The level of sulfane sulphur in the experimental groups. The experiments were carried out in gastric mucosa homogenates in three experimental group (Intact, WRS, WRS + NaHS). The data represent the mean value from two independent experiments. Each value is the mean of 8–20 repeats. Statistical analysis was performed using the Student’s *t*-test (* *p* < 0.05).

**Figure 3:**
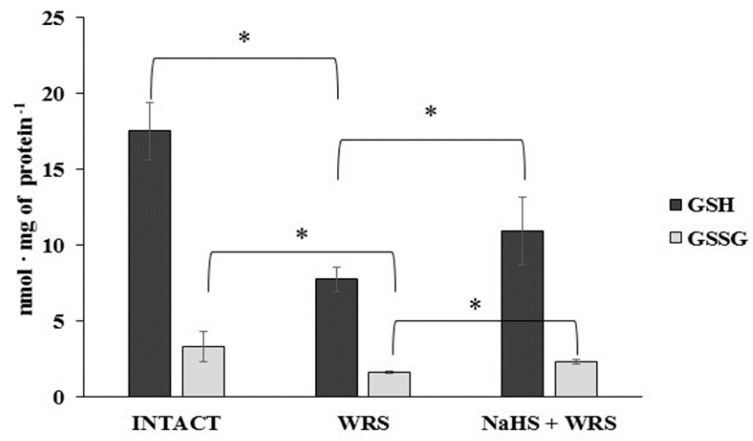
The level of reduced (GSH) and oxidized (GSSG) glutathione in the experimental groups. The experiments were carried out in gastric mucosa homogenates in three experimental group (Intact, WRS, WRS + NaHS) using the reversed-phase high-performance liquid chromatography (RP-HPLC) method. The data represent the mean value from two independent experiments. Each value is the mean of 8–10 repeats. Statistical analysis was performed using the Student’s *t*-test (* *p* < 0.05).

H_2_S is produced in the gastric mucosa of healthy rats (Figure 1). In the presence of l-cysteine, 2.32 ± 0.05 nmol of H_2_S is produced per 1 g of tissue during 1 h. It has also been postulated that H_2_S can be generated by degradation of persulfide, i.e., sulfane sulphur may be a precursor to biological H_2_S in the presence of thiols [10,11]. The determined level of sulfane sulphur in the gastric mucosa of healthy rats was 160 ± 60 nmol per 1 mg of protein (Figure 2). The GSH level equalled 17.5 ± 1.91 nmol/mg and the level of oxidized glutathione (GSSG)—3.35 ± 1.02 nmol/mg. Thus, the GSH/GSSG concentration ratio was 5.5 (Figure 3).

Table 1 shows the activity and expression of four enzymes involved in both H_2_S and sulfane sulphur metabolism (Scheme 1). The results confirm the expression of MPST, rhodanese, CTH and CBS in the gastric mucosa of healthy rats. The highest specific activity expressed in nmol of product produced during 1 min per 1 mg of protein was determined for MPST. On the other hand, non-detectable activity of CBS was found.

**Figure 4:**
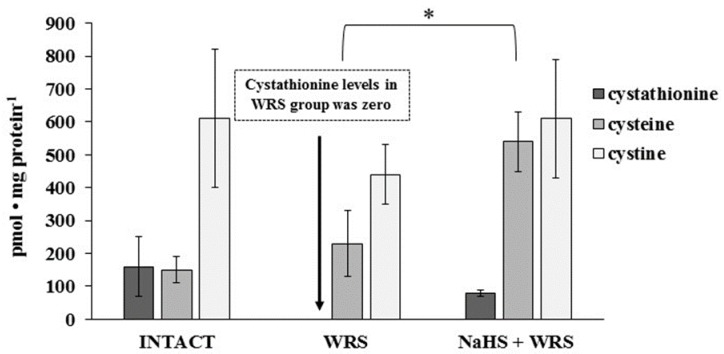
The level of cystathionine, cysteine, and cystine in the experimental group. The experiments were carried out in gastric mucosa homogenates in three experimental group using the RP-HPLC method. The data represent the mean value from two independent experiments. Each value is the mean of 8–10 repeats. Statistical analysis was performed using the Student’s *t*-test (* *p* < 0.05).

Cysteine, cystine, and cystathionine levels were also measured in the gastric mucosa of healthy rats and are presented in Figure 4. The total cysteine level in the tissue calculated as the sum of pmol of cysteine/mg and 2× pmol of cystine/mg equalled 1.4 nmol/mg.

On the basis of these results it can be concluded that substrates for MPST, CTH, CBS, and rhodanese are available in healthy gastric mucosa and the ability to generate H_2_S was confirmed in the tissue [4,12]. Mard et al. [4] and Martin et al. [12] reported the expression of CBS and CTH in the rat gastric mucosa and suggested CTH as the main enzyme responsible for H_2_S production. Based on the obtained results, it seems possible that due to the low activity of CBS and CTH, the main H_2_S generating pathway in the tissue involves MPST and 3-merceptopiruvate as a substrate (Scheme 1). The importance of rhodanese and sulfane sulphur can also be considered—the role of this enzyme in creation of H_2_S in tissues is the least studied [6].

### 2.2. Gastric Mucosa from Rats with Ulcers Induced by Water Immersion and Restraint Stress

The gastric mucosa of rats with ulcers induced by immersion in cold water (21 °C) for 3.5 h had a 2–3-fold higher ability to generate H_2_S in comparison to the gastric mucosa of healthy rats (Figure 1). The level of sulfane sulphur was similar to that in the healthy mucosa (Figure 2). The GSH and GSSG levels were significantly decreased as compared to the healthy mucosa (Figure 3) and the concentration ratio of GSH/GSSG was also slightly decreased. These results may suggest that a higher capability of H_2_S generation by ulcerated mucosa results from an increased specific activity of the enzymes involved in the process rather than from its increased release from sulfane sulphur stores. As it is shown in Table 1, no increased expression (messenger RNA (mRNA) levels) of the investigated enzymes was found in comparison to the healthy mucosa; however, changes in the specific activity were significant. A three-fold increased activity of CTH and a detectable CBS activity was noted in the WRS group. Similarly, the activity of MPST and rhodanese were also significantly increased. Accelerated cystathionine conversion by CTH resulted in its decreased level (Figure 4). Thus, stimulation of all enzyme-dependent pathways for H_2_S generation (Scheme 1), in response to stress resulting from water immersion and restraint, was confirmed. The results show that the defence mechanism against stress is associated with stimulation of the production of hydrogen sulphide in the tissue, and confirm the observed advantageous effect of H_2_S on healing of gastric ulcers [8,13,14].

### 2.3. NaHS Pretreatment Affects the Formation of Hydrogen Sulphide in Gastric Mucosa of Rats with WRS Induced Ulcers

The rats administered NaHS, a precursor of hydrogen sulphide, 30 min prior to WRS, demonstrated a decreased ability of endogenous H_2_S generation in gastric mucosa as compared to the gastric mucosa of rats with ulcers induced by immersion in cold water, not pre-administered with NaHS (Figure 1). Figure 2 shows a significantly higher level of sulfane sulphur in the gastric mucosa of rats pre-administered NaHS. This suggests that H_2_S released from NaHS results in an in-creased level of sulfane sulphur-containing compounds in the tissue [15]. The expression of all the investigated enzymes is not changed (Table 1) in the mucosa pre-treated with NaHS as compared to the gastric mucosa with ulcers induced by immersion in cold water in rats not pre-treated with NaHS. In contrast to the CTH activity, which was not affected by NaHS pre-treatment, the activity of CBS and rhodanese was found to be decreased and MPST increased.

Stress caused by immersion in cold water resulted in an increased activity of MPST and CTH in the ulcerated gastric mucosa, which remained high, independently of pre-treatment with an exogenous (NaHS) source of H_2_S, whereas NaHS pre-treatment resulted in a decrease in the activity of CBS and rhodanese to the level characteristic of the healthy mucosa.

The levels of GSH and GSSG in the case of NaHS pre-treatment were significantly higher in comparison to the ulcerated mucosa (Figure 3). They were reversed to the levels found in the healthy mucosa. A similar tendency was observed in the case of the level of cystathionine, cysteine, and cystine (Figure 4).

Thus, in comparison to the healthy mucosa, the changes observed in the ulcerated gastric mucosa, such as an increased production of H_2_S, an increased activity of the investigated enzymes, a decreased level of GSH, GSSG, and cystathionine, revert back to the levels/activities similar to these found in the healthy mucosa. These results confirm the beneficial effect of NaHS, as a donor of H_2_S, in changing some biochemical parameters (except MPST and CTH activities and the level of cysteine), back to the values found in the healthy tissue. This is in accordance with the previously mentioned gastroprotective effect of endogenous H_2_S and H_2_S released from the donors (NaHS) [8,13,14]. The mechanism by which H_2_S protects the gastric mucosa may involve an increase in gastric flow—the exposure of rats to 3.5 h of WRS causes gastric lesions and a significantly decreased gastric blood flow [16]. The results presented in this paper show that the defence against WRS-induced gastric mucosal lesions includes the acceleration of endogenous H_2_S formation. In case of pre-administration of an exogenous source of H_2_S, the changes observed in the gastric mucosa tend to maintain the activities of some mucosal enzymes involved in H_2_S generation and the levels of their substrates at levels characteristic to the healthy mucosa.

## 3. Material and Methods

### 3.1. Chemicals

l-Glutathione reduced, d,l-cystathionine (CTN), d,l-homoserine (HSer), 1-fluoro-2,4-dinitrobenzene (DNFB), bathophenanthroline-disulfonic acid disodium salt (BPDS), acetonitrile, PLP, β-nicotinamide adenine dinucleotide reduced disodium salt hydrate (NADH), l-lactic dehydrogenase (LDH), 3-mercaptopyruvate acid sodium salt, d,l-dithiothreitol, (DTT), *N*-ethylmaleimide (NEM), dl-propargylglycine (PPG), sodium dihydrogen phosphate dihydrate pure, sodium sulphite, chloroform, isopropanol, agarose, sodium hydrosulphide hydrate, sodium chloride, Folin-Ciocalteu’s phenol reagent, iron (III) nitrate nonahydrate, sodium thiosulfate pentahydrate, sodium carbonate, *N*,*N*-dimethyl-*p*-phenylenediamine sulphate salt, and sodium thiosulfate were obtained from Sigma-Aldrich (St. Louis, MO, USA). Trifluoroacetic acid (TFA) and 2-mercaptoethanol were purchased from Fluka Chemie GmbH (Buchs, Switzerland). Ethanol and 70% perchloric acid (PCA), 38% formaldehyde, 65% nitric acid, 38% hydrochloric acid, ammonia solution 25% pure, potassium sodium tartrate tetrahydrate, copper sulphate pentahydrate, potassium dihydrogen phosphate, ferric chloride, zinc acetate dehydrate pure, trichloroacetic acid (TCA), and sodium hydroxide were from Polskie Odczynniki Chemiczne S.A. (Gliwice, Poland). *N^ε^*-methyllysine was obtained from Bachem (Bubendorf, Switzerland). Trizol, ethidium bromide and EDTA (Ethylenediaminetetraacetic acid)—disodium salt dihydrate were obtained from Lab-Empire (Rzeszow, Poland). Potassium cyanide was from Merck (Darmstadt, Germany). Reverse transcriptase M-MuLV was obtained from Promega (Madison, WI, USA). Polymerase DNA Dream Taq™, Gene Ruler 100 bp DNA Ladder, Oligo (dT)_18_ primer and Deoxynucleotide (dNTP) Solution Mix were obtained from Abo (Gdańsk, Poland).

### 3.2. Animals

Male Wistar rats (220–300 g) were used in the experiments. They were deprived of food for 24 h with free access to tap water before the experiments. After the experiment, the rats were kept under anesthesia under pentobarbital (60 mg/kg, intraperitoneally (i.p.)) and the stomachs were removed. The stomach was slit along the curvatura major. Mucosal specimens were scraped off using a slide glass and immediately frozen in liquid nitrogen and stored at −80 °C until analysis as described in Magierowska et al. [17]. All of the experiments were conducted in cooperation with the Department of Physiology, Faculty of Medicine at the Jagiellonian University and were approved by the Institutional Animal Care and Use Committee of the Jagiellonian University Medical College in Cracow (No.: 68/2014) and performed in accordance with the Helsinki Declaration.

### 3.3. Experimental Group

**Scheme 2:**
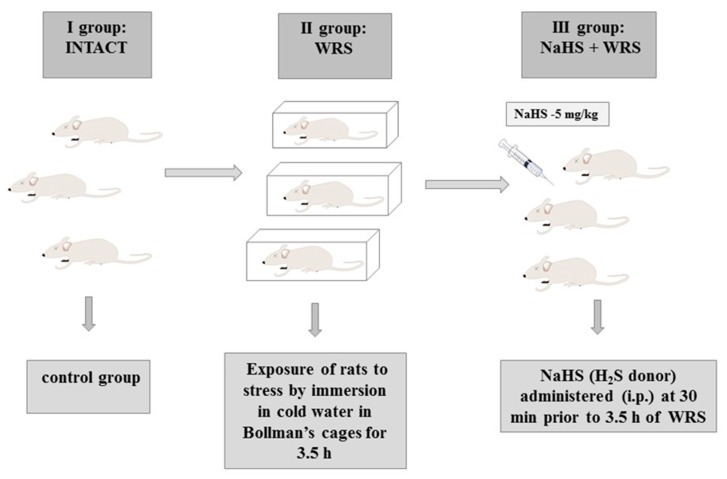
Experimental group. i.p.: intraperitoneally.

Stress lesions were caused by immobilizing the rats in individual Bollman’s cages and immersing the animals in cold water (21 °C) for 3.5 h as described in previous studies [18]. The experiment was carried out in three experimental groups: (1) the control group (intact); (2) vehicle (saline)-pre-treated 30 min prior to 3.5 h of water immersion and restraint stress (WRS); or (3) NaHS (H_2_S donor) administered i.p. at a dose of 5 mg/kg (Scheme 2).

### 3.4. Tissue Homogenates

For determinations of the enzyme activities (CTH, MPST, CBS, rhodanese) and the level of sulfane sulphur, the gastric mucosa samples were weighed and homogenized in ice-cold 0.1 M phosphate buffer pH 7.5 (1 g/4 mL) for 1 min at 8000–9500 rpm using a blender homogenizer. The homogenates were centrifuged at 1600 g for 10 min. After centrifugation, the supernatants were used for the determination of the enzyme activities (CTH, MPST, CBS rhodanese) and the level of sulfane sulphur and protein content.

For the reversed-phase high-performance liquid chromatography (RP-HPLC) the tissues were weighed and homogenized at 8000–9500 rpm in ice-cold 10% PCA/1 mM BPDS (1 g/3 mL) (1 g tissue/3 mL solution). The homogenates were centrifuged for 10 min at 4 °C at 1400× *g*. The supernatants were used for assays immediately or stored at −80 °C until HPLC analysis. The tissues were homogenized using an Ultra-Turrax T 25 (Janke and Kunkel IKA-Labortechnik Company, Staufen, Germany). The homogenates were centrifuged using a MPW 375 centrifuge (MPW MED Instruments, Warszawa, Poland) or a Hettich Universal 16 centrifuge (Hettich AG, Kloten, Switzerland).

### 3.5. RP-HPLC

The RP-HPLC method of Dominick et al. [19] with modifications [20,21,22] was used for the detection and quantitation of the levels of direct and non-related products of the CBS- and CTH-catalysed reactions, such as cystathionine, reduced (GSH) and oxidized (GSSG) glutathione, cysteine, and cystine.

### 3.6. Determination of H*_2_*S in the Homogenate of the Gastric Mucosa

**Scheme 3:**
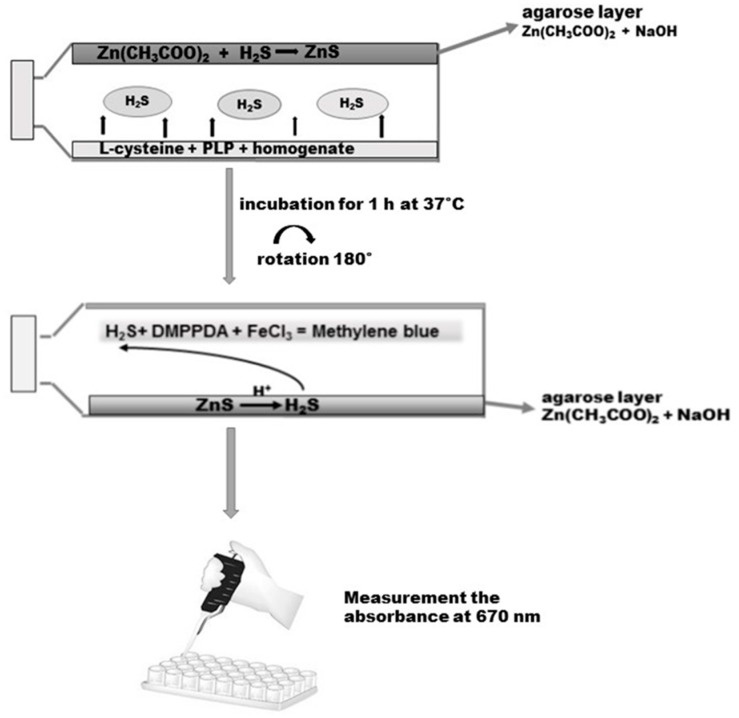
Determination of H_2_S in the homogenate of the gastric mucosa according to Kartha et al. [23]. Details are described in Materials and Methods. DMPPDA: *N*,*N*-dimethyl-*p*-phenylenediamine sulphate salt.

The gastric mucosa tissue samples were homogenized at a ratio of 1/8 with 50 mM phosphate buffer, pH 8.0. Then, the homogenates were incubated for 5 min at 37 °C on ice. Before the experiment, 50 mL tissue culture flasks with unventilated caps were covered with a layer of agarose mixed with 1% of zinc acetate and 3 M sodium hydroxide as described by Karth et al. [23]. On the opposite side of the layer of agarose, 5000 µL of a reaction mixture containing 4500 µL homogenate, 250 µL 2 mM of pyridoxal phosphate, and 250 µL of 10 mM l-cysteine (final concentration) was added. The caps of the flasks were secured with parafilm. The incubation was initiated by the transfer of the bottles with ice to 37 °C. After the 90-min incubation, 2500 µL 50% TCA was added to the reaction mixture. Another 60 min was allowed for the trapping of evolved H_2_S by the layer of agarose. After incubation the reaction mixture was removed from the flasks, the flasks were rotated through 180 °C and the reaction was conducted on the layer of agarose by adding 2 mL of 40 mM *N*,*N*-dimethyl-*p*-phenylenediamine sulphate salt (DMPPDA) and subsequently incubating for 10 min at room temperature, followed by addition of 400 µL 1% ferric chloride and re-incubation for 20 min at room temperature (Scheme 3). As a result of the reaction, methylene blue was formed, which was spectrophotometrically determined at 670 nm. The standard curve was linear over the concentration range of 0–250 µM with a correlation coefficient of 0.994.

### 3.7. Enzymes Assay in the Gastric Mucosa Homogenates

The MPST activity was assayed according to the method of Valentine and Frankelfeld [24], following a procedure described in our earlier paper [25]. The incubation mixture contained in a final volume of 500 µL: 250 µL, 0.12 M sodium phosphate buffer, pH 8.0, 50 µL, 0.5 M sodium sulfite, 50 µL 0.15 M dithiothreitol, 50 µL homogenates, 50 µL H_2_O, and 50 µL 0.1 M 3-mercaptopyruvate acid sodium salt. The mixture was incubated for 15 min. To stop the reaction, 250 µL of 1.2 M PCA was added. The samples were centrifuged at 1600× *g* for 5 min, and 100 µL of supernatant was transferred to 1350 µL mixture that contained: 1200 µL, 0.12 M sodium phosphate buffer, pH 8.0, 100 µL 0.1 M *N*-ethylmaleimide, and 50 µL NADH 5 mg/mL. After equilibration at 37 °C, 2.5 µL of l-lactic dehydrogenase (7 IU) were added, and the decrease in absorbance at 340 nm was measured. The enzyme activity was expressed as nmol of pyruvate produced during 1 min incubation at 37 °C per 1 mg of protein.

The γ-cystathionase activity was determined by the Matsuo and Greenberg’s method [26] with modifications described by Czubak et al. [27]. The incubation mixture contained: 25 µL 1.3 mM PLP, 25 µL 13 mM EDTA, 250 µL 45 mM cystathionine solution in 0.1 M phosphate buffer, pH 7.5 (2.5 mg cystathionine per sample), 75 µL homogenates, and 0.1 M phosphate buffer, pH 7.5 containing 0.05 mM 2-mercaptoethanol, in a final volume of 650 µL. The reaction was stopped after 15 min of incubation at 37 °C by placing 125 µL incubation mixture in 25 µL 10% PCA. The samples were centrifuged at 1600× *g* for 10 min, and 25 µL of supernatant was transferred to 625 µL 0.194 mM NADH solution and kept at 37 °C. The control samples, without 45 mM cystathionine, were prepared in the same way as the examined samples. After 10 s of the measurement (absorbance at 340 nm), 25 µL (9.06 IU) l-lactic dehydrogenase were added and the measurement was continued to 180 s. The difference between the initial value of absorbance (before addition of LDH) and the lowest value (after adding LDH) corresponded to the amount of α-ketobutyrate formed in the course of the γ-cystathionase reaction. The γ-cystathionase activity is expressed as nmol of α-ketobutyrate formed.

Sulfane sulphur was determined by the method of Wood [28], based on cold cyanolysis and colorimetric detection of the ferric thiocyanate complex ion. Incubation mixtures in a final volume 880 µL contained: 20 µL 1 M ammonia solution, 20 µL homogenate, 740 µL H_2_O, and 100 µL 0.5 M sodium cyanide. The incubation was performed for 45 min at room temperature. After incubation, thiocyanate was estimated calorimetrically at 460 nm after the addition of 20 µL 38% formaldehyde and 40 µL ferric nitrate reagent. The sulfane sulphur level is expressed as nmol of SCN^−^ (thiocyanate) produced per 1 mg of protein during 1 min incubation at 37 °C per 1 mg of protein.

The activity of CBS was examined in homogenates in the presence of d,l-homoserine (HSer) as a substrate. After 15 min of the incubation at 37 °C, the methods described in Bronowicka-Adamska et al. [21] were used. The level of cystathionine was determined using the HPLC method described by Bronowicka-Adamska et al. [20]. The CBS activity is expressed as pmol of cystathionine formed during 1 min incubation at 37 °C per 1 mg of protein.

Rhodanese in the gastric mucosa homogenates was assayed according to Sörbo [29] with modifications. Incubation mixtures in a final volume 500 µL contained: 200 µL, 0.125 M sodium thiosulfate, 100 µL, 0.2 M potassium phosphate (KH_2_PO_4_), 100 µL, 0.25 M sodium cyanide, and 100 µL homogenate. The incubation was performed during 5 min at 20 °C, after which thiocyanate was estimated colorimetrically at 460 nm after the addition of 20 µL of 38% formaldehyde and 40 µL ferric nitrate reagent. The enzyme units are defined as µmol of SCN^−^ generated per minute per 1 mg of protein at 20 °C under the prescribed assay conditions.

The protein concentration was determined by the method of Lowry et al. [30] using crystalline bovine serum albumin as a standard.

### 3.8. Expression of MPST, CTH, and CBS in the Gastric Mucosa Homogenates

#### 3.8.1. RNA Extraction

Total RNA was extracted using TRIzol (Lab-Empire S.A (Rzeszow, Poland)), according to the protocol provided by the manufacturer. The quality of the RNA samples was determined by spectrophotometric analysis (A_260_/A_280_) and electrophoresis in 2.5% agarose gel followed by staining with ethidium bromide.

#### 3.8.2. Reverse Transcription of RNA

Total RNA from the cell samples was reverse-transcribed using a First-Stand complementary DNA (cDNA) synthesis kit according to the manufacturer instructions (Promega, Madison, WI, USA). For reverse transcription (RT), 3 µg of the total RNA was mixed with 1 µL of Oligo (dT)_15_ (0.5 µg/reaction) and nuclease-free water and heated in a 70 °C heat block for five minutes. After pre-incubation, the reverse transcription reaction mix containing: 4 µL GoScript 5× reaction buffer (Promega, Madison, WI, USA), 3µL MgCl_2_ (final concentration 1.5–5.0 mM), 1 µL dNTPs (10 mM), 1 µL Recombinant RNases Ribonuclease Inhibitor (20 U/µL), and 1 µL GoScript Reverse Transcriptase was prepared.

#### 3.8.3. cDNA Synthesis and RT-PCR Analysis

**Figure 5:**
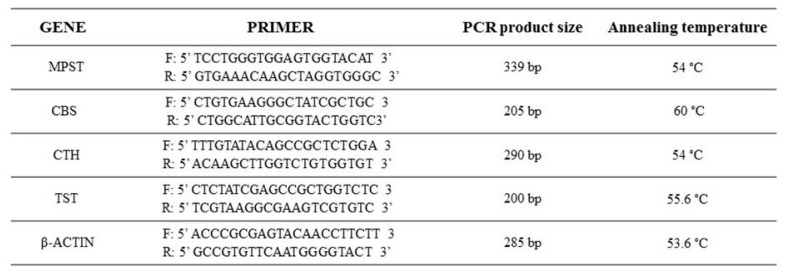
Forward and reverse primers used in the RT-PCR reaction to assess messenger RNA (mRNA) expression for MPST, CBS, CTH, TST, and β-actin.

Expressions of MPST, CTH, CBS, rhodanese, and β-actin were analysed by RT-PCR. Amplification of cDNA samples was run in a 12.5 µL reaction volume containing 1 µL of synthesized cDNA, 0.2 µM of each of the gene-specific primer pair, 0.04 U/µL DNA polymerase in 10 mM buffer Tris–HCl pH 8.8, 0.2 mM each of dNTPs and nuclease-free water. The temperature profile of RT-PCR amplification for MPST consisted of activation of Taq DNA polymerase (Abo, Gdansk, Poland) at 94 °C for 5 min, denaturation of cDNA at 95 °C for 30 s, primer annealing at 54 °C for 30 s, elongation at 72 °C for 1 min for the following 28 cycles, and finishing by the extension step for 8 min. For the *CTH* gene, after an initial 5 min at 94 °C denaturation, amplification was performed under the following conditions: 95 °C for 30 s, 56 °C for 30 s, and 72 °C for 2 min for 36 cycles, with a final incubation at 72 °C for 8 min. For the *CBS* gene [4], after an initial 10 min of denaturation at 95 °C, amplification was performed under the following conditions: 94 °C for 20 s, 60 °C for 1 min, and 72 °C for 1 min for 40 cycles, with a final incubation at 72 °C for 5 min. For the *β-actin* gene, after an initial 5 min denaturation at 94 °C, amplification was performed under the following conditions: 94 °C for 30 s, 53.6 °C for 30 s, and 72 °C for 2 min for 28 cycles, with a final incubation at 72 °C for 8 min. For the *rhodanese* gene, after an initial 5 min denaturation at 94 °C, amplification was performed under the following conditions: 94 °C for 30 s, 55.6 °C for 30 s, and 72 °C for 2 min for 38 cycles, with a final incubation at 72 °C for 8 min. The specific primers (Oligo.pl, Warszawa, Poland) were used (Figure 5). The PCR reaction products were separated electrophoretically in a 2.5% agarose gel, visualized with ethidium bromide, directly visualized under ultraviolet (UV) light, and photographed.

### 3.9. Statistical Analysis

All results are expressed as means ± SEM (standard error of the mean). The significance of the differences between the controls and the investigated samples was calculated using the Student’s *t*-test (*p* < 0.05) (MS Excel 2013). Each experiment was repeated a minimum of three times.

## 4. Conclusions

Endogenous synthesis of H_2_S is stimulated in the gastric mucosa as a compensatory mechanism to damage induced by WRS. Hydrogen sulphide is produced in the gastric mucosa in response to injury and acts to promote healing when its precursor, NaHS, is administered prior to WRS. The results suggest that H_2_S-releasing drugs could be employed to accelerate healing of gastric ulcers.

## Figures and Tables

**Table 1:** The activity and expression of MPST, rhodanese, CTH, and CBS in the experimental group.

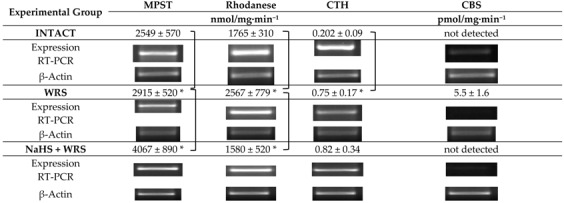

The experiments were carried out in gastric mucosa homogenates in three experimental group. The data represent the mean value from two independent experiments. Each value is the mean of 10–15 repeats. Statistical analysis was performed using the Student’s *t*-test (* *p* < 0.05). MPST, TST (rhodanese), CTH, and CBS gene expression levels were evaluated by RT-PCR (reverse transcription polymerase chain reaction) analysis. One set of representative results is shown. β-Actin was used as an internal control.
